# Enteral Lactoferrin Supplementation for Preventing Sepsis and Necrotizing Enterocolitis in Preterm Infants: A Meta‑Analysis With Trial Sequential Analysis of Randomized Controlled Trials

**DOI:** 10.3389/fphar.2020.01186

**Published:** 2020-08-07

**Authors:** Ya Gao, Liangying Hou, Cuncun Lu, Qi Wang, Bei Pan, Quan Wang, Jinhui Tian, Long Ge

**Affiliations:** ^1^ Evidence-Based Medicine Center, School of Basic Medical Sciences, Lanzhou University, Lanzhou, China; ^2^ Key Laboratory of Evidence-Based Medicine and Knowledge Translation of Gansu Province, Lanzhou, China; ^3^ Evidence-Based Social Science Research Center, School of Public Health, Lanzhou University, Lanzhou, China; ^4^ Gansu Provincial Hospital, Lanzhou, China; ^5^ Department of Gastrointestinal Surgery & Laboratory of Surgical Oncology, Peking University People’s Hospital, Peking University, Beijing, China; ^6^ Department of Social Medicine and Health Management, School of Public Health, Lanzhou University, Lanzhou, China

**Keywords:** lactoferrin, preterm infant, sepsis, necrotizing enterocolitis, mortality, meta‑analysis, trial sequential analysis

## Abstract

**Background:**

Several clinical trials investigated the effects of enteral lactoferrin supplementation on the prevention of sepsis and necrotizing enterocolitis (NEC) in preterm infants, but the efficacy and safety remain disputed. Therefore, we systematically evaluated the effect of enteral lactoferrin supplementation in preterm infants through a meta‑analysis with trial sequential analysis (TSA).

**Methods:**

We searched six databases to identify randomized controlled trials (RCTs) that evaluated the effects of lactoferrin supplementation compared with placebo or no intervention in preterm infants. RevMan version 5.3 software was used to estimate pooled relative risks (RRs) with the random-effects model. TSA, subgroup analyses, and meta-regression analyses were also performed.

**Results:**

Nine RCTs with 3515 samples were included. With low to moderate quality of evidence, compared with placebo, enteral lactoferrin supplementation did not significantly decrease the incidences of late-onset sepsis (RR = 0.63, 95% CI: 0.38 to 1.02, *P* = 0.06), NEC stage II or III (RR = 0.68, 95% CI: 0.30 to 1.52, *P* = 0.35), all-cause mortality (RR = 0.89, 95% CI: 0.51 to 1.57, *P* = 0.69), bronchopulmonary dysplasia (RR = 1.01, 95% CI: 0.90 to 1.13, *P* = 0.92), retinopathy of prematurity (RR = 0.80, 95% CI: 0.49 to 1.32, *P* = 0.38), invasive fungal infection (RR = 0.27, 95% CI: 0.02 to 3.94, *P* = 0.34), intraventricular hemorrhage (RR = 1.40, 95% CI: 0.39 to 5.08, *P* = 0.61), and urinary tract infection (RR = 0.35, 95% CI: 0.11 to 1.06, *P* = 0.06). Subgroup analysis revealed that lactoferrin significantly reduced the incidence of sepsis in infants with a birth weight below 1500 g (RR = 0.43, 95% CI: 0.22 to 0.84, *P* = 0.01). TSAs of the primary outcomes showed that the evidence is insufficient and further data is required.

**Conclusions:**

Limited evidence suggested that enteral lactoferrin supplementation was associated with a reduction of late-onset sepsis in infants with a birth weight below 1500g, however, did not decrease the incidence of NEC stage II or III, all-cause mortality, and other adverse events in preterm infants. The present evidence was insufficient to inform clinical practice.

## Introduction

Complications of preterm birth were the leading cause of death in children under five worldwide, accounting for 35% of neonatal death (Liu et al., 2012; Chawanpaiboon et al., 2019). The late-onset infection (occurring >72 hours after birth) is the most common serious complication associated with hospital care for preterm infants (Kaufman and Fairchild, 2004; ELFIN Trial Investigators Group, 2019), which can increase the risk of mortality and acute morbidities, including necrotizing enterocolitis, retinopathy of prematurity, and bronchopulmonary dysplasia, especially it is always associated with worse neurodevelopment (Stoll et al., 2004; Stoll et al., 2005; Bassler et al., 2009; Shane et al., 2017). Also, it requires invasive procedures and hospital healthcare with a great finical and social burden (Manzoni et al., 2010;  ELFIN trial investigators group, 2019). Therefore, the prevention of late-onset infection in preterm infants is extremely paramount.

Lactoferrin is a member of the iron-binding glycoprotein transferrin family, as well as a component of the innate immune response and an effective immunomodulator in mammals (Wu et al., 2014; Legrand, 2016; ELFIN Trial Investigators Group, 2019). It is the main whey protein in human milk, with a high concentration in human colostrum, up to 9 mg/ml (Ronayne De Ferrer et al., 2000), and a concentration of about 1 mg/ml in mature milk (ELFIN Trial Investigators Group, 2019). It can also be found in human tears, saliva, and semen (Pammi and Suresh, 2017). Lactoferrin can promote the growth of probiotics, stimulate the differentiation and proliferation of intestinal cells and the expression of intestinal digestive enzymes, thereby exerting anti-inflammatory effects (Raghuveer et al., 2002; Legrand, 2016; ELFIN Trial Investigators Group, 2019). Lactoferrin also has antibacterial, antiviral, antifungal, anticancer, and immunomodulatory properties (Kell et al., 2020). Therefore, it is often used to prevent late-onset infections and other morbidities.

A Cochrane review (Pammi and Suresh, 2017) published in 2017 identified six randomized controlled trials (RCTs) enrolling 1041 preterm infants, which concluded that lactoferrin supplementation reduced the risk of late-onset infection by 40% and necrotizing enterocolitis by 60%. However, this finding was low quality of evidence owing to limited sample size and poor quality of primary studies (Pammi and Suresh, 2017). Recently, however, a large, international, multicenter RCT (the ELFIN trial) (ELFIN Trial Investigators Group, 2019) involving 2182 preterm infants has been published, which concluded that enteral supplementation with bovine lactoferrin did not reduce the risk of late-onset infection in very preterm infants. Therefore, a new meta-analysis is necessary to supplement and update the evidence on enteral lactoferrin supplementation for prevention of late-onset infection.

This systematic review and meta-analysis aimed to incorporate the latest data and systematically assess the effect of lactoferrin supplementation on preterm infants and conduct a trial sequential analysis (TSA) to determine the optimal sample size, as well as rate the quality of evidence using GRADE (Grades of Recommendation, Assessment, Development, and Evaluation) approach.

## Methods

### Study Registration

We registered the protocol of this study on international prospective register of systematic review (PROSPERO) (CRD42019123163) and reported the full-text according to the Preferred Reporting Items for Systematic Reviews and Meta-Analyses (PRISMA) statement (Moher et al., 2009).

### Search Strategy

PubMed, the Cochrane Central Register of Controlled Trials (CENTRAL), EMBASE.com, Chinese Biomedical Literature Database (CBM), China National Knowledge Infrastructure (CNKI), and Wanfang Database were searched from their inception to January 22, 2019, and we updated the search on August 17, 2019. The search terms used included “infant,” “neonate,” “premature,” “low birth weight,” “lactoferrin,” and “RCTs”. The detailed search strategy for each database is presented in **Text S1**. We manually reviewed reference lists of eligible studies and relevant systematic reviews.

### Eligibility Criteria

We included RCTs that compared the effect of lactoferrin supplementation versus placebo or no intervention for preventing infection in preterm (<37 completed weeks of gestation) neonates (<28 days). There were no restrictions on the dosage and duration of lactoferrin, publication language, and publication status. Eligible RCTs should report at least one of the outcomes of interest. We excluded RCTs focusing on premature infants with severe congenital abnormalities such as chromosomal abnormalities, gastrointestinal malformations, milk allergy, or anticipated intestinal fasting for more than 14 days. We also excluded abstracts because they did not provide enough information for assessing the quality of study (Tan et al., 2019).

### Outcomes

The primary outcomes were (i) confirmed sepsis during the hospital stay. Confirmed sepsis was defined as clinical signs and symptoms consistent with infection and microbiologically proven with positive blood culture, cerebrospinal fluid culture, urine culture, or culture from a normally sterile site (e.g., pleural fluid, peritoneal fluid, and autopsy specimens) for bacteria or fungi (Pammi and Suresh, 2017). (ii) Necrotizing enterocolitis (NEC) Bell’s stage II or III (definitive NEC and perforated NEC, Bell’s stage II or III) (Bell et al., 1978) during hospital stay. (iii) “All-cause mortality” and “sepsis-attributable mortality” during the hospital stay. The secondary outcomes were (i) bronchopulmonary dysplasia, (ii) retinopathy of prematurity, (iii) invasive fungal infection, (iv) intraventricular hemorrhage, and (v) urinary tract infection.

### Screening and Data Extraction

We used EndNote X8 software to manage the retrieved records. Two reviewers (Y.G. and L.Y.H.) independently screened the title and abstract of each record according to eligibility criteria and reviewed the full-texts of potentially relevant studies. Disagreements were resolved by discussion or consultation of a third reviewer (L.G). A standardized data abstracted form was established using Microsoft Excel 2016 (Microsoft Corp, Redmond, WA; www.microsoft.com) to collect the following information: study characteristics (first author, year of publication, and study design), population characteristics (gestational age, birth weight, sex, and sample size), interventions details, and outcomes of interest. Two reviewers (Y.G. and L.Y.H.) independently abstracted the data, and conflict was resolved by discussion.

### Risk of Bias Assessment

Two reviewers (YG and LH) independently assessed the risk of bias of individual study according to the Cochrane Risk of Bias tool (Higgins et al., 2011), which included randomization sequence generation, allocation concealment, blinding of participants and personnel, blinding of outcome assessment, incomplete outcome data, selective reporting, and other sources of bias (baseline imbalances and funding support). We graded each item as low risk, high risk, or unclear risk. Conflicts were resolved by discussion.

### Certainty of the Evidence

We rated the certainty (quality) of evidence using the GRADE approach that classified evidence as high, moderate, low, and very low certainty (Salanti et al., 2014). The start point for RCTs was high but may be rated down because of serious study limitation, serious inconsistency, serious imprecision, serious indirectness, and serious publication bias.

### Data Synthesis

Review Manager (RevMan) version 5.3 (The Nordic Cochrane Centre, Rigshospitalet, Copenhagen, Denmark) was used to estimate pooled relative risks (RRs) and their 95% confidence intervals (CIs) for dichotomous outcomes using the Mantel-Haenszel statistical method with the random-effects model. We assessed statistical heterogeneity in each pairwise comparison with I^2^ statistic, and the value of <25%, 26–50%, and >50% considered as low, moderate, and high level of heterogeneity, respectively (Higgins et al., 2003).

To determine whether the current sample size is sufficient in our meta-analysis and to prevent repeated updates from increasing the risk of random errors, we conducted trial sequential analyses (TSAs) using TSA software (version 0.9 Beta; Copenhagen Trial Unit, Copenhagen, Denmark) (Bangalore et al., 2011; Xue et al., 2019). The TSA was performed with a 5% risk of type I error, 20% risk of type II error, and power of 80%. We estimated the required information size using a random-effects model based on an RR reduction of 20%, and the event proportions of control group calculated from the included trials (Wetterslev et al., 2008; Brok et al., 2009; Gu et al., 2016).

We planned to conduct subgroup analyses based on the following subgroup factors: gestational age (<32 weeks vs. ≥32 weeks), birth weight (<1500g vs. ≥1500 g), and feedings (breast milk vs. formula milk). We conducted within study subgroup analyses if there were at least two trials in each subgroup (Wang et al., 2018). Sensitivity analyses were performed by excluding trials with high risk or unknown risk of bias of the different domains for the primary outcomes and also by excluding the largest sample trial. We used univariate meta-regression to assess if either the primary outcomes or the heterogeneity was associated with the region of patients and dose of lactoferrin. We conducted Egger’s test using Stata (version 13.0; StataCorp) to detect publication bias. All statistic significant threshold was set at *P* < 0.05 with two-tailed.

## Results

### Identification of Relevant Studies

**Figure 1:**
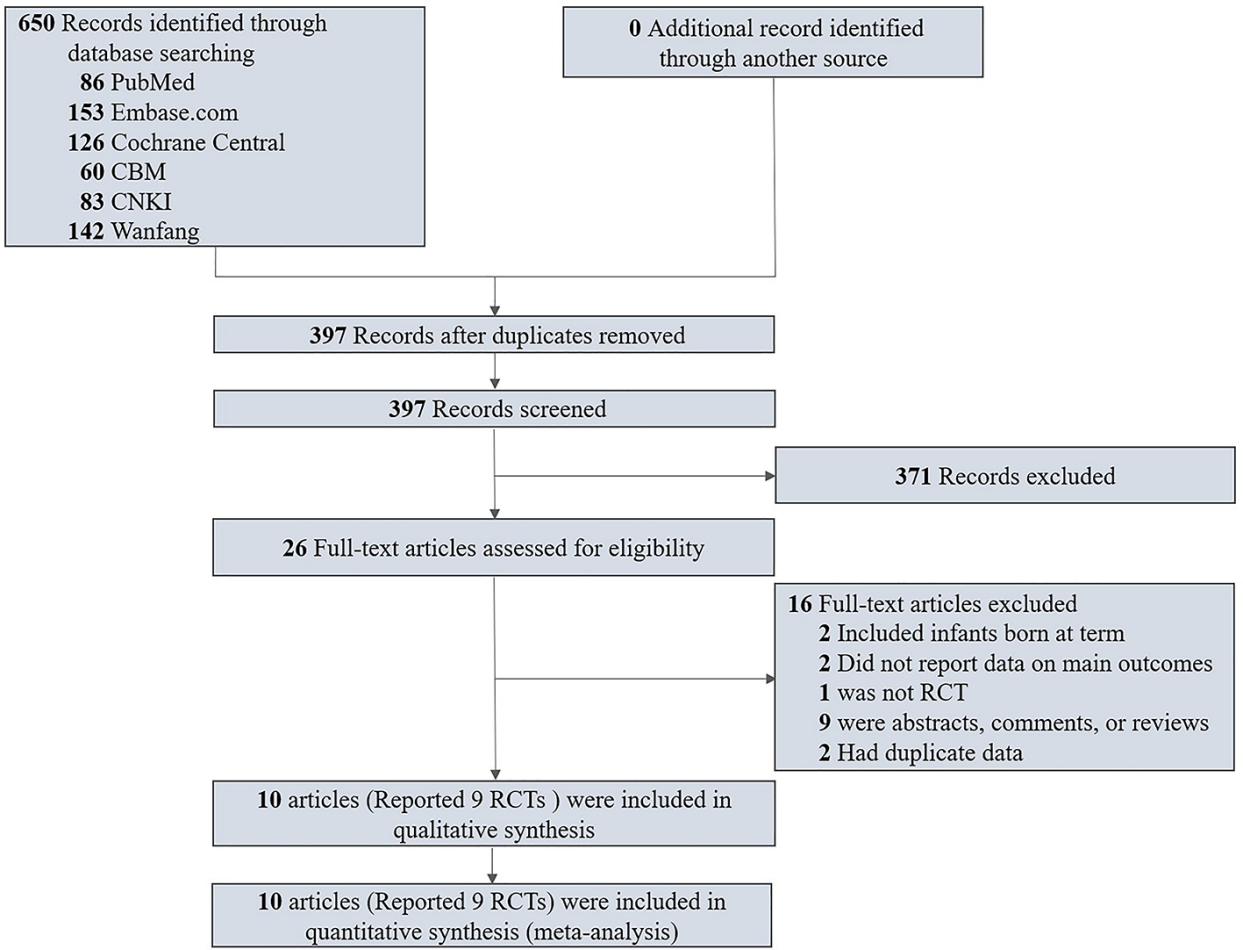
Flow Diagram of Study Selection.

The search yielded 650 records, among which 365 were from English databases and 285 were from Chinese databases. After reviewing the titles and abstracts, 371 records were excluded, and 26 were identified for full-text review. Finally, 10 articles (nine RCTs) (Manzoni et al., 2009; Akin et al., 2014; Manzoni et al., 2014; Dai and Xie, 2015; Kaur and Gathwala, 2015; Ochoa et al., 2015; Barrington et al., 2016; Sherman et al., 2016; Tang et al., 2017; ELFIN Trial Investigators Group, 2019) proved eligible (**Figure 1**).

### Characteristics of Included Studies

Nine RCTs were published in between 2014 and 2019, enrolled patients between 2007 and 2017, and located in Turkey, Canada, China, the United Kingdom, India, Italy, New Zealand, Peru, and the United States. The sample size of participants per study ranged from 50 to 2,199 (a total of 3,515). Types of lactoferrin included bovine lactoferrin (nine articles) (Manzoni et al., 2009; Akin et al., 2014; Manzoni et al., 2014; Dai and Xie, 2015; Kaur and Gathwala, 2015; Ochoa et al., 2015; Barrington et al., 2016; Tang et al., 2017; ELFIN Trial Investigators Group, 2019) and talactoferrin (one article) (Sherman et al., 2016). The start time of lactoferrin supplementation for included studies was within the first 72 hours of preterm infant life. Eight RCTs diluted lactoferrin in prepared milk or formula, and one RCT used the talactoferrin solution. The dose of lactoferrin ranged from 100 to 300 mg/day. Types of microorganisms that caused sepsis included methicillin-resistant coagulase-negative *Staphylococcus*, *Enterococcus*, *Escherichia coli*, *Klebsiella*, *Pseudomonas*, *Candida*, *Acinetobacter baumanii*, *Enterobacter aerogenes*, and group B *Streptococcus*. The detailed characteristics of the included studies are summarized in **Table 1**.

**Table 1:** Characteristics of included studies.

Study	Language	Setting	Study period	Sample	Sex (M/F)	Gestational age (weeks)		Birth weight (g)		Study group			Start time; duration	Route of lactoferrin used	Types of microorganisms that cause sepsis
						Intervention	Control	Intervention	Control	Intervention	Control	Dose			
Akin et al., 2014	English	Turkey	2009.12–2011.1	50	23/27	29.5 ± 1.6	30.3 ± 2.5	1290 ± 346.7	1307 ± 262.1	BLF	Placebo	200 mg/day	Within the first 72 h of life; until death or discharge	Oral, LF was diluted in milk or formula	MRCONS, *Enterococcus*, *Escherichia coli*, *Klebsiella pneumonia*, *Pseudomonas*, *Candida*
Barrington et al., 2016	English	Canada	2012.12–2013.9	79	46/33	28.0 ± 1.7	28.4 ± 2.1	1087 ± 315	1104 ± 320	BLF	Placebo	100 mg/day	Within the first 48 h of life; until 36 weeks post-menstrual age or discharge	Enteral, LF was diluted in milk or formula	CONS, *Escherichia* *coli*, *Klebsiella*, *Candida*
Dai and Xie, 2015	Chinese	China	2010.10–2014.5	70	NR	30.03 ± 2.16	30.03 ± 2.16	<1500	<1500	BLF	Placebo	100 mg/day	Within the first 72 h of life; NR	NR	NR
ELFIN Trial Investigators Group, 2019	English	United Kingdom	2014.5–2017.9	2199	1153/1046	29 (IQR 27–30)	29(IQR 27–30)	1125.9 ± 356.2	1143.3 ± 367.1	BLF	Placebo	150 mg/kg/day	Within the first 72 h of life; until death or discharge	Enteral, LF was diluted in milk or formula	NR
Kaur and Gathwala, 2015	English	India	2012.5–2013.6	130	73/57	34.4 ± 2.9	33.9 ± 2.5	1494.98 ± 240.87	1484.01 ± 224.86	BLF	Placebo	100–250 mg/day	Within the first 12 h of life; until 28th day of life	Enteral, LF was diluted in milk or formula	*Pseudomonas aeruginosa*, *Acinetobacter baumanii*, *Klebsiella*, *Staphylococcus aureus*, *Escherichia coli*, *Candida*
Manzoni et al., 2009; Manzoni et al., 2014	English	Italy, New Zealand	2007.10–2010.7	505	264/241	29.7 ± 2.5	29.4 ± 3.1	1158 ± 251	1118 ± 259	BLF	Placebo	100 mg/day	Within the first 72 h of life; until day 30 (45 for neonates <1,000 g at birth)	Enteral, LF was diluted in milk or formula	NR
Ochoa et al., 2015	English	Peru	2011.1–2011.8	190	92/98	32.2 ± 2.6	32.0 ± 2.6	1582 ± 422	1600 ± 395	BLF	Placebo	200 mg/kg/day	Within the first 72 h of life; until day 30.	Enteral, LF was diluted in milk or formula	*Serratia*, *Enterobacter aerogenes*, *Klebsiella*, CONS, *Pseudomonas*, Group B *Streptococcus*, *Enterococcus faecalis*
Sherman et al., 2016	English	United States	2009.7–2012.3	120	69/51	28 ± 6/7	28 ± 6/7	1152 ± 206	1143 ± 220	TLF	Placebo	300 mg/kg/day	Within the first 24 h of life; until 28th day of life or discharge	Oral, TLF solution	NR
Tang et al., 2017	Chinese	China	2013.1–2015.12	172	78/94	31.63 ± 2.50	31.55 ± 3.28	1542.34 ± 244.12	1509.17 ± 269.24	BLF	Placebo	100 mg/day	Within the first 72 h of life; until death or discharge	Enteral, LF was diluted in milk or formula	NR

M, male; F, female; NR, not report; IQR, interquartile range; BLF, bovine lactoferrin; TLF, talactoferrin; LF, lactoferrin; MRCONS, methicillin-resistant coagulase-negative staphylococcus; CONS, methicillin-resistant coagulase-negative staphylococcus.

### Risk of Bias and Quality of Evidence

The risk of bias of included studies is presented in **Figures S1** and **S2**. Three RCTs (Akin et al., 2014; Dai and Xie, 2015; Tang et al., 2017) did not provide sufficient information about the random sequence generation process and allocation concealment. Two RCTs (Dai and Xie, 2015; Tang et al., 2017) did not clarify the blinding of participants and personnel. Only two RCTs (Akin et al., 2014; Ochoa et al., 2015) described the blinding of outcome assessment, and other biases of the four RCTs (Dai and Xie, 2015; Kaur and Gathwala, 2015; Sherman et al., 2016; Tang et al., 2017) were unclear. The certainty of evidence evaluated by GRADE is shown in **Table 2**. The certainty of the evidence was rated as moderate or low for all the outcomes, most often because of the insufficient sample size.

**Table 2:** Summary of findings.

GRADE profile for assessing the quality of evidence						
Patient or population: preterm infantsSetting: neonatal intensive care unitsIntervention: Oral lactoferrinComparison: placebo						
Outcomes	Anticipated absolute effects^*^ (95% CI)		Relative effect(95% CI)	№ of participants(studies)	Certainty of the evidence(GRADE)	Comments
	Risk with control	Risk with oral lactoferrin				
Sepsis - All infants	160 per 1,000	**101 per 1,000** (61 to 163)	**RR 0.63** (0.38 to 1.02)	3310 (9 RCTs)	⨁⨁◯◯ LOW ^a,b^	
NEC ≥ stage II	53 per 1,000	**36 per 1,000** (16 to 81)	**RR 0.68** (0.30 to 1.52)	2919 (5 RCTs)	⨁⨁◯◯ LOW ^b,c^	
Mortality - all-cause mortality	61 per 1,000	**54 per 1,000** (31 to 96)	**RR 0.89** (0.51 to 1.57)	3265 (7 RCTs)	⨁⨁◯◯ LOW ^b,c^	
Mortality - sepsis-attributable mortality	48 per 1,000	**11 per 1,000** (1 to 82)	**RR 0.24** (0.03 to 1.72)	813 (4 RCTs)	⨁⨁⨁◯ MODERATE ^b^	
Bronchopulmonary dysplasia	285 per 1,000	**288 per 1,000** (257 to 322)	**RR 1.01** (0.90 to 1.13)	2638 (4 RCTs)	⨁⨁⨁◯ MODERATE ^b^	
Retinopathy of prematurity	75 per 1,000	**60 per 1,000** (37 to 99)	**RR 0.80** (0.49 to 1.32)	2748 (4 RCTs)	⨁⨁⨁◯ MODERATE ^b^	
Invasive fungal infection	58 per 1,000	**16 per 1,000** (1 to 229)	**RR 0.27** (0.02 to 3.94)	493 (2 RCTs)	⨁⨁⨁◯ MODERATE ^b^	
Intraventricular hemorrhage	53 per 1,000	**75 per 1,000** (21 to 270)	**RR 1.40** (0.39 to 5.08)	509 (2 RCTs)	⨁⨁◯◯ LOW ^a,b^	
Urinary tract infections	66 per 1,000	**23 per 1,000** (7 to 70)	**RR 0.35** (0.11 to 1.06)	440 (2 RCTs)	⨁⨁⨁◯ MODERATE ^b^	
*The risk in the intervention group (and its 95% confidence interval) is based on the assumed risk in the comparison group and the **relative effect** of the intervention (and its 95% CI). **CI,** Confidence interval; **RR,** Risk ratio						
**GRADE Working Group grades of evidence** **High certainty:** We are very confident that the true effect lies close to that of the estimate of the effect **Moderate certainty:** We are moderately confident in the effect estimate: The true effect is likely to be close to the estimate of the effect, but there is a possibility that it is substantially different **Low certainty:** Our confidence in the effect estimate is limited: The true effect may be substantially different from the estimate of the effect **Very low certainty:** We have very little confidence in the effect estimate: The true effect is likely to be substantially different from the estimate of effect						

^a^Moderate or severe heterogeneity (>50% heterogeneity).

^b^Total sample size is lower than the optimal information size. ^c^Point estimates are inconsistent.

### Primary Outcomes

**Figure 2:**
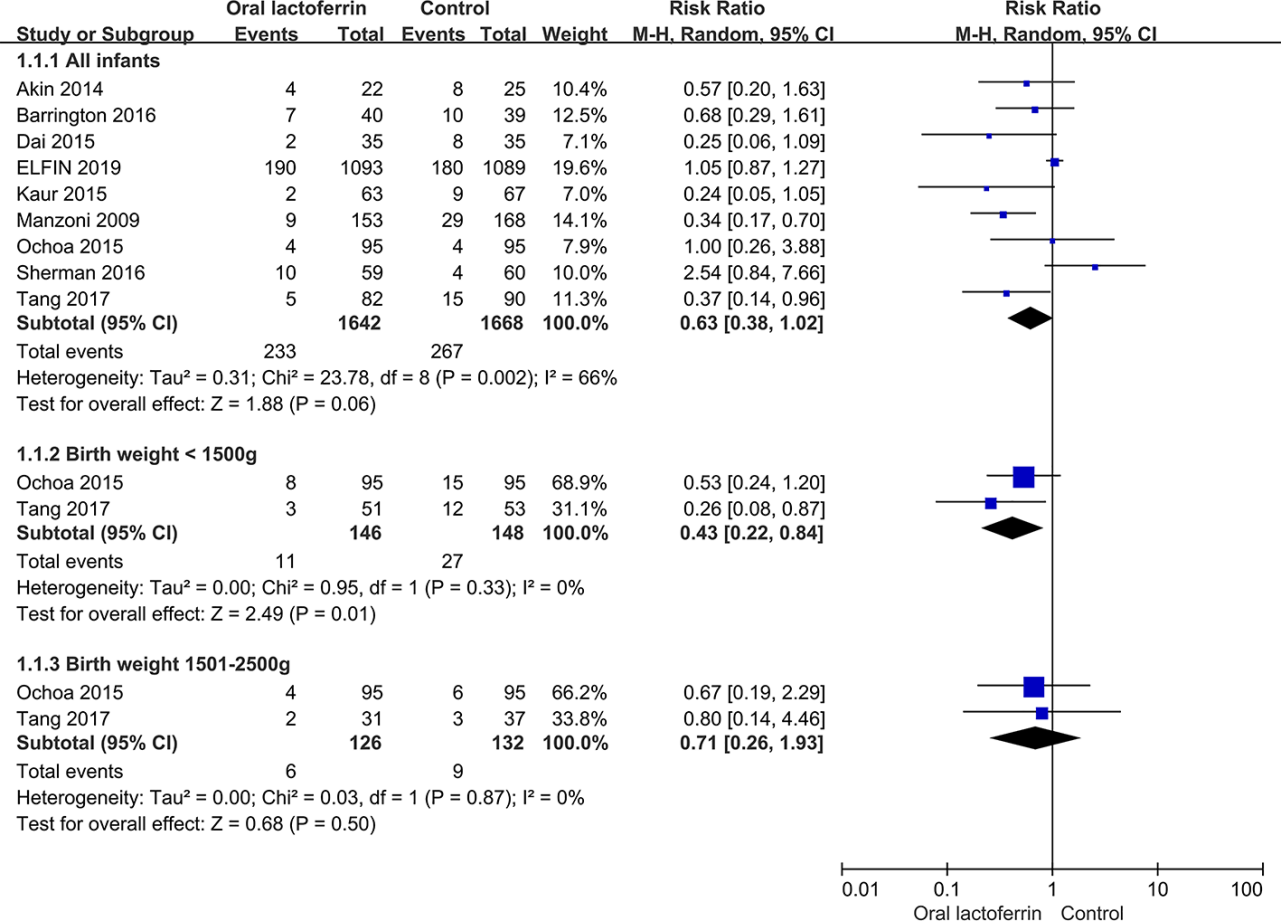
Comparison of the incidence of sepsis between enteral lactoferrin supplementation and placebo.

**Figure 3:**
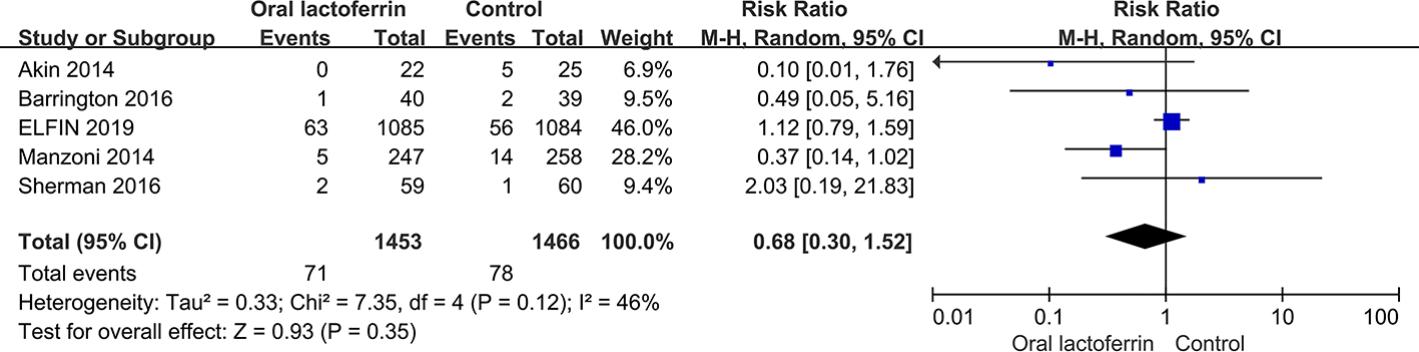
Comparison of the incidence of NEC stage II or III between enteral lactoferrin supplementation and placebo.

**Figure 4:**
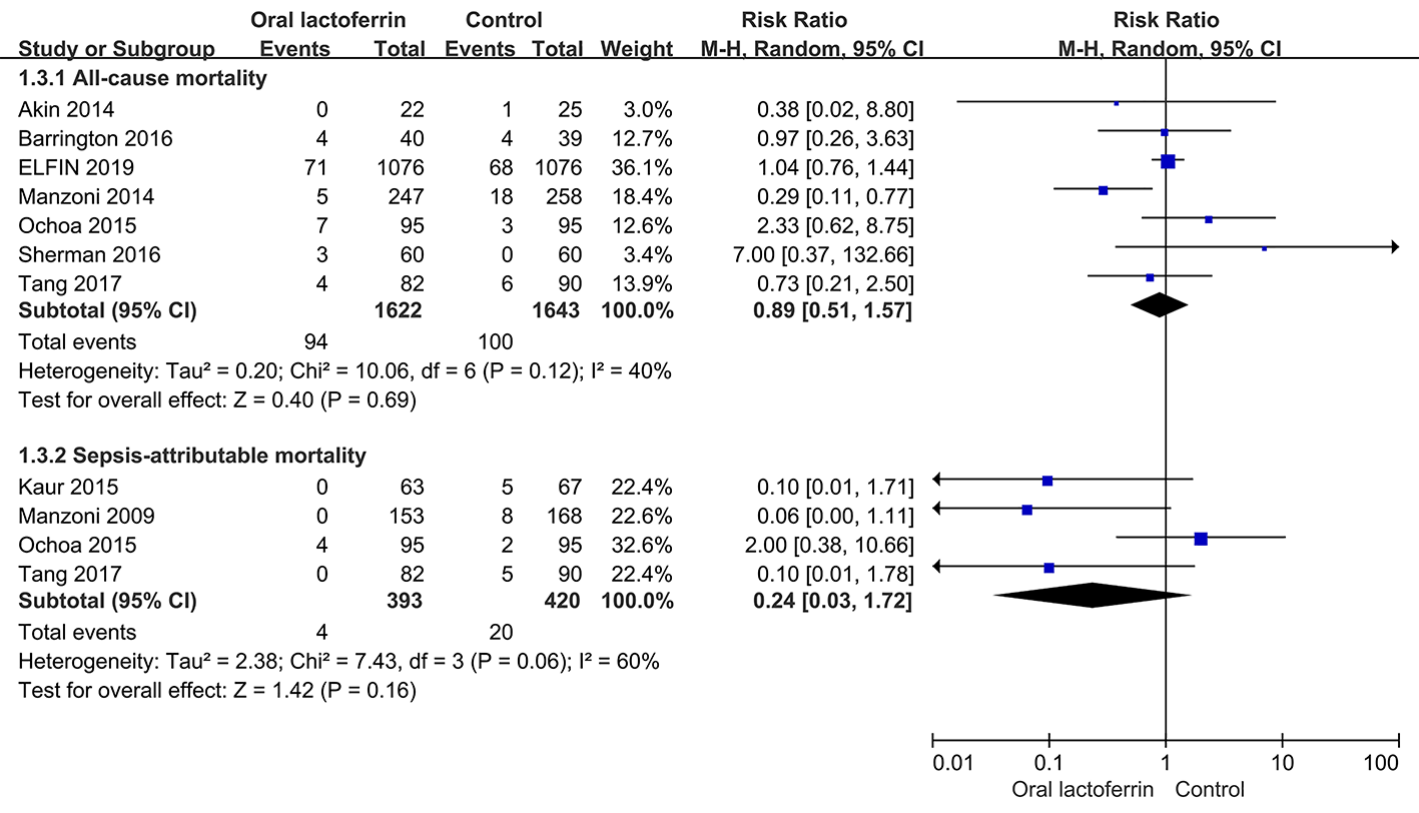
Comparison of mortality between enteral lactoferrin supplementation and placebo.

With low to moderate quality of evidence, there were no significant differences between enteral lactoferrin supplementation and placebo in the incidences of late-onset sepsis [nine RCTs (Manzoni et al., 2009; Akin et al., 2014; Dai and Xie, 2015; Kaur and Gathwala, 2015; Ochoa et al., 2015; Barrington et al., 2016; Sherman et al., 2016; Tang et al., 2017; ELFIN Trial Investigators Group, 2019), 3,310 patients; RR = 0.63, 95% CI: 0.38 to 1.02, *P* = 0.06, I^2^ = 66%; low certainty] (**Figure 2**), NEC stage II or III [five RCTs (Akin et al., 2014; Manzoni et al., 2014; Barrington et al., 2016; Sherman et al., 2016; ELFIN Trial Investigators Group, 2019), 2,919 patients; RR = 0.68, 95% CI: 0.30 to 1.52, *P* = 0.35, I^2^ = 46%; low certainty] (**Figure 3**), all-cause mortality [seven RCTs (Akin et al., 2014; Manzoni et al., 2014; Ochoa et al., 2015; Barrington et al., 2016; Sherman et al., 2016; Tang et al., 2017; ELFIN Trial Investigators Group, 2019), 3,265 patients; RR = 0.89, 95% CI: 0.51 to 1.57, *P* = 0.69, I^2^ = 40%; low certainty], and sepsis-attributable mortality [four RCTs (Manzoni et al., 2009; Kaur and Gathwala, 2015; Ochoa et al., 2015; Tang et al., 2017), 813 patients; RR = 0.24, 95% CI: 0.03 to 1.72, *P* = 0.16, I^2^ = 60%; moderate certainty] (**Figure 4**).

### Secondary Outcomes

**Figure 5:**
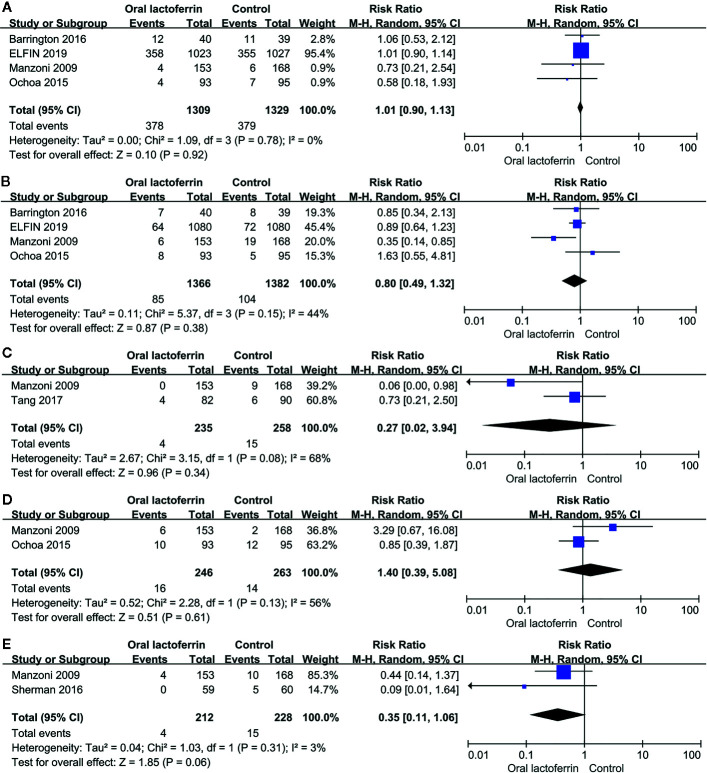
Comparisons of the incidence of **(A)** bronchopulmonary dysplasia, **(B)** retinopathy of prematurity, **(C)** invasive fungal infection, **(D)** intraventricular hemorrhage, and **(E)** urinary tract infection between enteral lactoferrin supplementation and placebo.

Compared with placebo, enteral lactoferrin supplementation did not significantly decrease the incidences of bronchopulmonary dysplasia [four RCTs (Manzoni et al., 2009; Ochoa et al., 2015; Barrington et al., 2016; ELFIN Trial Investigators Group, 2019), 2,638 patients; RR = 1.01, 95% CI: 0.90 to 1.13, *P* = 0.92, I^2^ = 0%; moderate certainty] (**Figure 5A**), retinopathy of prematurity [four RCTs (Manzoni et al., 2009; Ochoa et al., 2015; Barrington et al., 2016; ELFIN Trial Investigators Group, 2019), 2,748 patients; RR = 0.80, 95% CI: 0.49 to 1.32, *P* = 0.38, I^2^ = 44%; moderate certainty] (**Figure 5B**), invasive fungal infection [two RCTs (Manzoni et al., 2009; Tang et al., 2017), 493 patients; RR = 0.27, 95% CI: 0.02 to 3.94, *P* = 0.34, I^2^ = 68%; moderate certainty] (**Figure 5C**), intraventricular hemorrhage [two RCTs (Manzoni et al., 2009; Ochoa et al., 2015), 509 patients; RR = 1.40, 95% CI: 0.39 to 5.08, *P* = 0.61, I^2^ = 56%; low certainty] (**Figure 5D**), and urinary tract infection [two RCTs (Manzoni et al., 2009; Sherman et al., 2016), 440 patients; RR = 0.35, 95% CI: 0.11 to 1.06, *P* = 0.06, I^2^ = 3%; moderate certainty) (**Figure 5E**).

### Trial Sequential Analyses

We performed trial sequential analyses to explore whether cumulative data for primary outcomes were adequate. TSAs showed that the cumulative z curve crossed the conventional significance test boundary but did not reach the trial sequential monitoring boundary for benefit and the estimated information size boundary, indicating the evidence is insufficient and more trials are needed (**Figures S3**–**S4**, **S5**).

### Subgroup Analyses

We conducted subgroup analyses for the primary outcomes. In subgroup analysis by birth weight (<1500 g vs. ≥1500 g), the pooled results indicated that enteral lactoferrin supplementation significantly reduces the incidence of late-onset sepsis in infants with a birth weight below 1500g (RR = 0.43, 95% CI: 0.22 to 0.84, *P* = 0.01, I^2^ = 0%) (**Figure 2**). There was no significant difference between enteral lactoferrin supplementation and placebo in the incidence of sepsis in infants with a birth weight between 1501 and 2500 g (RR = 0.71, 95% CI: 0.26 to 1.93, *P* = 0.50, I^2^ = 0%) (**Figure 2**). However, no significant subgroup effect was found (*P*_interaction_ = 0.41). Due to limited data, we were unable to perform other predefined subgroup analyses on the outcomes.

### Sensitivity Analyses and Meta-Regression Analyses

Sensitivity analyses showed that the effects of enteral lactoferrin supplementation on sepsis, NEC stage II or III, and all-cause mortality did not change substantially after excluding studies with high or unknown risk of bias of the different domains. However, the enteral lactoferrin supplementation significantly decreased the incidence of late-onset sepsis (RR = 0.55, 95% CI: 0.33 to 0.92, *P* = 0.02) and NEC stage II or III (RR = 0.42, 95% CI: 0.19 to 0.97, *P* = 0.04) after removing the largest trial (ELFIN Trial Investigators Group, 2019), although the effect on all-cause mortality (RR = 0.86, 95% CI: 0.37 to 1.97, *P* = 0.72) was similar to the overall analysis (**Table S1**). Univariate meta-regression analyses showed that the region of patients and dose of lactoferrin were not the source of heterogeneity or the key factors affecting the overall effect size (*P* > 0.05) (**Figures S6**–**S7**, **S8**).

### Publication Bias

Egger’s tests indicated no significant publication bias in items of late-onset sepsis (*P* = 0.091), NEC stage II or III (*P* = 0.279), and all-cause mortality (*P* = 0.922).

## Discussion

This study systematically evaluated the effect of enteral lactoferrin supplementation in preterm infants. A comprehensive search of six electronic databases was performed, and eventually, we included nine RCTs. Our meta-analyses indicated that enteral lactoferrin supplementation was not associated with a reduction in late-onset sepsis in all infants. However, subgroup analysis within studies showed that enteral lactoferrin supplementation significantly reduced the incidence of late-onset sepsis in very low birth weight (VLBW) and extremely low birth weight (ELBW) infants. Compared with placebo, enteral lactoferrin supplementation did not decrease the incidence of NEC stage II or III, bronchopulmonary dysplasia, retinopathy of prematurity, invasive fungal infection, intraventricular hemorrhage, and urinary tract infection. Besides, there were no differences in all-cause mortality and sepsis-related mortality between the enteral lactoferrin supplement group and the placebo group.

Previous systematic reviews and meta-analyses (Pammi and Suresh, 2017; He et al., 2018) have assessed the safety and effectiveness of lactoferrin supplementation to enteral feeds for the prevention of sepsis and NEC in preterm infants. These studies revealed that lactoferrin supplementation to enteral feeds with or without probiotics could significantly decrease the incidence of late-onset sepsis, NEC stage II or III, risk of hospital-acquired infection, and infection-related mortality in preterm infants without obvious adverse effects (Pammi and Suresh, 2017; He et al., 2018). However, our meta-analysis indicated that enteral lactoferrin supplementation did not reduce the incidence of sepsis, NEC stage II or III, all-cause mortality, sepsis-related mortality, and other adverse outcomes, which were inconsistent with previous meta-analyses. Therefore, differences between our study and previous meta-analyses should be noted. Compared to previous studies, our research has several advantages that make it more conclusive. First, our present meta-analysis included 9 trials involving a total of 3515 samples compared to no more than 1834 samples in previous meta-analyses. Thus, the present study had enlarged sample sizes and added statistical power of at least 1600 cases. Second, to explore whether the current evidence was reliable and conclusive, and to prevent repeated updates from increasing the risk of random errors, we further applied TSA to evaluate the effects of repetitive testing, which increased the robustness of our findings (Huang et al., 2018; Huang et al., 2020). Third, we rated the quality of evidence using GRADE approach with the guidance of an experienced GRADE methodological expert. We found that the quality of evidence was low to moderate compared to the low quality of evidence in the Cochrane review, which was the only one conducted GRADE assessment. The reasons for rating down the quality between these two reviews were different. In our review, we did not consider the domain of blinding of outcomes assessment in assessing study limitations because we considered that all outcomes of interest were objective. We rated down the study limitation only when the weight of high risk of bias studies was more than the weight of low risk of bias studies. The most often reason for rating down was the serious imprecision. Because we found that the sample sizes for all outcomes did not reach the optimal sample sizes according to our TSA results. However, in Cochrane review, the most often reason for rating down was serious study limitation and did not rate down because of serious imprecision for all outcomes. Fourth, our study assessed more clinical outcomes, such as retinopathy of prematurity and intraventricular hemorrhage, which had not been evaluated in previous reviews. Finally, in addition to conducting subgroup analyses to evaluate the impact of different factors on the effect of enteral lactoferrin supplement, we also performed sensitivity analyses and meta-regression analyses. Furthermore, we conducted Egger’s test to detect publication bias, and the results suggested there was no publication bias.

Lactoferrin can inhibit the growth of bacteria, fungi, viruses, and protozoa through cell membrane disruption, iron chelation, immunomodulation and synergy with anti-infectives, exhibiting a wide range of microbicidal activities (Wakabayashi et al., 2006; Zuccotti et al., 2007; Embleton et al., 2013). Studies have shown that lactoferrin was effective in preventing late-onset sepsis and neonatal necrotizing enterocolitis in preterm neonates (Walker, 2010; Pammi and Suresh, 2017). Our meta-analysis found that enteral lactoferrin supplementation could reduce the incidence of late-onset sepsis in VLBW and ELBW infants, although it was not associated with a reduction in late-onset sepsis in all infants. This may be related to a longer duration of treatment and greater cumulative dose for VLBW and ELBW infants. But the results indicated that there was no significant difference between the enteral lactoferrin supplementation and placebo in preventing the incidence of NEC stage II or III. However, when we conducted a sensitivity analysis by removing study with the largest sample size, the enteral lactoferrin supplementation could significantly decrease the incidence of sepsis and NEC stage II or III. Lactoferrin can form strong complexes with bacterial lipopolysaccharides to create a hole in the outer membrane of Gram-negative bacteria to exert bactericidal activity against many pathogens (Embleton and Berrington, 2020). Sequestration and binding of iron also prevent many pathogens from using their siderophores to obtain iron needed for growth (Oftedal, 2012). Lactoferrin is hydrolyzed under acidic conditions to produce a peptide called lactoferrin, which has been shown to have enhanced antimicrobial activity (Gifford et al., 2005; Pammi and Suresh, 2017). Lactoferrin creates an environment for the growth of beneficial bacteria in the gut, thereby reducing the colonization of pathogenic bacteria (Pammi and Suresh, 2017). Furthermore, lactoferrin also modulates cytokines or chemokines produced by gut-associated lymphoid tissue cells, regulates innate and acquired immune pathways, and promotes neurodevelopment and intestinal maturation in preterm infants (Tomita et al., 2002; Legrand, 2016; Pammi and Suresh, 2017). Therefore, lactoferrin may be beneficial for prophylaxis of neonatal preterm birth complications. TSA showed that the cumulative z curve crossed the conventional significance test boundary but reached neither the trial sequential monitoring boundary for benefit nor the estimated information size boundary, which may explain the unexpected result in our sensitivity analysis. Therefore, we were unable to establish sufficient and conclusive evidence that enteral lactoferrin supplementation did not affect the incidence of late-onset sepsis and NEC. Further trials are needed to validate our results.

Infection is a common cause of death in premature infants, with up to 1.6 million newborns dying every year worldwide (Kaufman and Fairchild, 2004; Manzoni et al., 2011). Although anti-infective drugs are currently available for the treatment of sepsis in preterm infants, the mortality rate of sepsis in preterm infants remains high (Stoll et al., 2002). However, the findings of our meta-analysis revealed that enteral lactoferrin supplementation did not affect all-cause mortality, which is consistent with the previous studies (Pammi and Suresh, 2017; He et al., 2018). Furthermore, the results also showed that lactoferrin had no significant benefit on the bronchopulmonary dysplasia, retinopathy of prematurity, invasive fungal infection, intraventricular hemorrhage, and urinary tract infection. Therefore, our study did not recommend that clinicians and parents use lactoferrin to prevent mortality or late-onset infection-associated adverse events in preterm infants.

Our study conducted a comprehensive literature search, detailed data collection and extraction, evaluated more clinical outcomes, and assessed the quality of evidence using the GRADE approach. Moreover, to improve the robustness of this meta-analysis, we applied TSA to assess the effects of random errors and repetitive tests (Gu et al., 2016). However, our study also had some limitations. First, the trials included in this review are small, although we searched six databases to incorporate all the eligible RCTs and manually searched the reference lists of relevant SRs to obtain additional trials. Second, since the outcomes we were concerned with are laboratory-confirmed indicators, the blinding of outcome assessors has little effect on the judgment of outcomes, thus we did not consider the domain of blinding of outcomes assessment when conducting the GRADE assessment. Third, we observed a high degree of heterogeneity between studies of certain outcomes that have not yet been fully resolved. Although we performed subgroup and sensitivity analyses to evaluate heterogeneity, some factors were not evaluated due to limited data. Fourth, a significant number of suspected cases of neonatal sepsis are early-onset sepsis, but the effectiveness of lactoferrin in preventing/treating early-onset sepsis was not reported in any of the included studies. Fifth, the certainty of the evidence was rated as moderate or low for all outcomes. Thus, the current findings should be interpreted with caution. TSA showed that the sample sizes of the main outcomes are insufficient, so large-scale, well-designed RCTs on this topic are still needed, and we cannot stop conducting trials with this compound in a specific subgroup (e.g., birth weight > 1500 g). Furthermore, more studies should focus on specific groups >1500 g with a higher risk (e.g., cardiac cases), more outcomes, or longer follow-up.

## Conclusions

Our meta-analysis suggested that enteral lactoferrin supplementation was associated with a reduction in late-onset sepsis in VLBW and extremely ELBW infants but did not decrease the incidence of NEC stage II or III, all-cause mortality, bronchopulmonary dysplasia, retinopathy of prematurity, invasive fungal infection, intraventricular hemorrhage, and urinary tract infection in preterm infants. However, due to the low certainty of evidence and small sample size, the conclusions were insufficient and inconclusive. More high-quality RCTs are needed to provide robust evidence of the effects of enteral lactoferrin supplementation in preterm infants, and we cannot stop conducting trials with this compound in a specific subgroup.

## Author Contributions

YG, LH, CL, and LG conceived the study protocol. YG, LH, CL, QiW, and LG participated in the literature search and the data collection. YG, BP, QuanW, JT, and LG analyzed the data. YG and LG drafted the manuscript. YG, JT, and LG revised the manuscript. All authors contributed to the article and approved the submitted version.

## Conflict of Interest

The authors declare that the research was conducted in the absence of any commercial or financial relationships that could be construed as a potential conflict of interest.

## Supplementary Material

Text S1 Search strategy. (DOCX 20kb).

Figure S1 Risk of bias about each risk of bias item resented as percentages across all included studies. (TIF 2356 kb).

Figure S2 Risk of bias summary for each included study. Yellow ()? indicates unclear risk; and green (+) indicates low risk of bias. (TIF 5643 kb).

Figure S3 Trial sequential analysis on pooled results of nine trials comparing enteral lactoferrin with placebo for the prevention of sepsis. Trial sequential analysis indicating that the cumulative z curve (blue) crossed the conventional line, but reached neither the trial sequential monitoring boundary for benefit nor the estimated information size boundary. A diversity-adjusted required information size of 33132 patients was calculated using α=0.05 (two-sided) and β=20 (power 80%), an anticipated relative risk reduction of 20%, and an event proportion of 16.01% in the control arm. (TIF 1444 kb).

Figure S4 Trial sequential analysis on pooled results of five trials comparing enteral lactoferrin with placebo for prevention of Necrotizing Enterocolitis (NEC) Bell’s stage II or III. Trial sequential analysis indicating that the cumulative z curve (blue) crossed the conventional line, but reached neither the trial sequential monitoring boundary for benefit nor the estimated information size boundary. A diversity-adjusted required information size of 44684 patients was calculated using α=0.05 (two-sided) and β=20 (power 80%), an anticipated relative risk reduction of 20%, and an event proportion of 5.32% in the control arm. (TIF 1414 kb).

Figure S5 Trial sequential analysis on pooled results of seven trials comparing enteral lactoferrin with placebo for reducing all-cause mortality. Trial sequential analysis indicating that the cumulative z curve (blue) crossed the conventional line, but reached neither the trial sequential monitoring boundary for benefit nor the estimated information size boundary. A diversity-adjusted required information size of 33269 patients was calculated using α=0.05 (two-sided) and β=20 (power 80%), an anticipated relative risk reduction of 20%, and an event proportion of 6.09% in the control arm. (TIF 1424 kb).

Figure S6 Univariate meta-regression analysis on the (A) region of patients, (B) dose of lactoferrin for the outcome of late-onset sepsis. (TIF 7069 kb).

Figure S7 Univariate meta-regression analysis on the dose of lactoferrin for the outcome of NEC stage II or III. (TIF 2950 kb).

Figure S8 Univariate meta-regression analysis on the (A) region of patients, (B) dose of lactoferrin for the outcome of all-cause mortality. (TIF 7005 kb).

Table S1 Results of sensitivity analysis. (XLS 12 kb).

## Abbreviations

RCT, randomized controlled trials; TSA, trial sequential analysis; GRADE, Grades of Recommendation, Assessment, Development, and Evaluation; NEC, necrotizing enterocolitis; RR, relative risk; CI, confidence interval.
