# Supplementary material for: Enteral Lactoferrin Supplementation for Preventing Sepsis and Necrotizing Enterocolitis in Preterm Infants: A Meta‑Analysis With Trial Sequential Analysis of Randomized Controlled Trials
Source: Front Pharmacol. 2020 Aug 7;11:1186. doi: 10.3389/fphar.2020.01186 (PMC7426497; doi:10.3389/fphar.2020.01186)
Supplement: Text S1 — Search strategy. (DOCX 20kb). [file DataSheet_1.docx]

**Additional file 1: Text S1. Search strategy**

**PubMed search strategy:**

#1 "Infant, Low Birth Weight"[Mesh] OR "Infant, Extremely Low Birth Weight"[Mesh] OR "Infant, Very Low Birth Weight"[Mesh] OR "Infant"[Mesh] OR "Infant, Premature"[Mesh] OR "Infant, Newborn"[Mesh] OR "Infant, Extremely Premature"[Mesh] OR "Premature Birth"[Mesh]

#2 Infant[Title/Abstract] OR Infants[Title/Abstract] OR neonate[Title/Abstract] OR neonates[Title/Abstract] OR neonatal[Title/Abstract] OR newborn[Title/Abstract] OR newborns[Title/Abstract] OR premature[Title/Abstract] OR preterm[Title/Abstract] OR "low birth weight"[Title/Abstract] OR "low birth weights"[Title/Abstract] OR LBW[Title/Abstract] OR ELBW[Title/Abstract] OR VLBW[Title/Abstract]

#3 #1 OR #2

#4 "Lactoferrin"[Mesh]

#5 lactoferrin[Title/Abstract] OR Lactotransferrin[Title/Abstract] OR talactoferrin[Title/Abstract]

#6 #4 OR #5

#7 "Clinical Trials, Phase II as Topic"[Mesh] OR "Clinical Trials, Phase III as Topic"[Mesh] OR "Clinical Trials, Phase IV as Topic"[Mesh] OR "Controlled Clinical Trials as Topic"[Mesh] OR "Randomized Controlled Trials as Topic"[Mesh] OR "Intention to Treat Analysis"[Mesh] OR "Pragmatic Clinical Trials as Topic"[Mesh] OR "Clinical Trials, Phase II"[Publication Type] OR "Clinical Trials, Phase III"[Publication Type] OR "Clinical Trials, Phase IV"[Publication Type] OR "Controlled Clinical Trials"[Publication Type] OR "Randomized Controlled Trials"[Publication Type] OR "Pragmatic Clinical Trials as Topic"[Publication Type] OR "Single-Blind Method"[Mesh] OR "Double-Blind Method"[Mesh]

#8 random*[Title/Abstract] OR blind*[Title/Abstract] OR singleblind*[Title/Abstract] OR doubleblind*[Title/Abstract] OR trebleblind*[Title/Abstract] OR tripleblind*[Title/Abstract]

#9 #7 OR #8

#10 #3 AND #6 AND #9

**Embase search strategy:**

#1 'newborn'/exp OR 'infant'/exp OR 'prematurity'/exp OR 'low birth weight'/exp OR 'very low birth weight'/exp OR 'extremely low birth weight'/exp

#2 'Infant':ab,ti OR 'Infants':ab,ti OR 'neonate':ab,ti OR 'neonates':ab,ti OR 'neonatal':ab,ti OR 'newborn':ab,ti OR 'newborns':ab,ti OR 'premature':ab,ti OR 'preterm':ab,ti OR 'low birth weight':ab,ti OR 'low birth weights':ab,ti OR 'LBW':ab,ti OR 'ELBW':ab,ti OR 'VLBW':ab,ti

#3 #1 OR #2

#4 'Lactoferrin'/exp

#5 'lactoferrin':ab,ti OR 'Lactotransferrin':ab,ti OR 'talactoferrin':ab,ti

#6 #4 OR #5

#7 'multicenter study (topic)'/exp OR 'phase 2 clinical trial (topic)'/exp OR 'phase 3 clinical trial (topic)'/exp OR 'phase 4 clinical trial (topic)'/exp OR 'controlled clinical trial (topic)'/exp OR 'randomized controlled trial (topic)'/exp OR 'single blind procedure'/exp OR 'double blind procedure'/exp

#8 random*:ab,ti OR blind*:ab,ti OR singleblind*:ab,ti OR doubleblind*:ab,ti OR trebleblind*:ab,ti OR tripleblind*:ab,ti

#9 #7 OR #8

#10 #3 AND #6 AND #9

**Cochrane Library search strategy:**

#1 MeSH descriptor: [Infant, Low Birth Weight] explode all trees

#2 MeSH descriptor: [Infant, Extremely Low Birth Weight] explode all trees

#3 MeSH descriptor: [Infant, Very Low Birth Weight] explode all trees

#4 MeSH descriptor: [Infant] explode all trees

#5 MeSH descriptor: [Infant, Premature] explode all trees

#6 MeSH descriptor: [Infant, Newborn] explode all trees

#7 MeSH descriptor: [Infant, Extremely Premature] explode all trees

#8 MeSH descriptor: [Premature Birth] explode all trees

#9 (Infant):ti,ab,kw OR (Infants):ti,ab,kw OR (neonate):ti,ab,kw OR (neonates):ti,ab,kw OR (neonatal):ti,ab,kw OR (newborn):ti,ab,kw OR (newborns):ti,ab,kw OR (premature):ti,ab,kw OR (preterm):ti,ab,kw OR ("low birth weight"):ti,ab,kw OR ("low birth weights"):ti,ab,kw OR (LBW):ti,ab,kw OR (ELBW):ti,ab,kw OR (VLBW):ti,ab,kw

#10 OR/#1-9

#11 MeSH descriptor: [Lactoferrin] explode all trees

#12 (lactoferrin):ti,ab,kw OR (Lactotransferrin):ti,ab,kw OR (talactoferrin):ti,ab,kw

#13 #11 OR #12

#14 #10 AND #13

**CBM search strategy:**

#1 "婴儿, 早产"[不加权:扩展] OR "婴儿, 极低出生体重"[不加权:扩展] OR "婴儿, 超低出生体重"[不加权:扩展] OR "婴儿, 出生时低体重"[不加权:扩展] OR "婴儿, 新生"[不加权:扩展]

#2 "早产儿"[常用字段] OR "新生儿"[常用字段] OR "未成熟儿"[常用字段] OR "婴儿"[常用字段] OR "低出生体重儿"[常用字段] OR "低体重儿"[常用字段] OR "极低出生体重儿"[常用字段] OR "极低体重儿"[常用字段] OR "超低出生体重儿"[常用字段] OR "超低体重儿"[常用字段] OR "低出生体质量儿"[常用字段] OR "低体质量儿"[常用字段] OR "极低出生体质量儿"[常用字段] OR "极低体质量儿"[常用字段] OR "超低出生体质量儿"[常用字段] OR "超低体质量儿"[常用字段]

#3 #1 OR #2

#4 "乳铁蛋白"[不加权:扩展]

#5 "乳铁蛋白"[常用字段] OR "乳铁传递蛋白"[常用字段] OR "乳铁转铁蛋白"[常用字段]

#6 #4 OR #5

#7 #3 AND #6

**CNKI search strategy:**

#1 SU=出生时低体重 OR SU=早产儿 OR SU=新生儿 OR SU=未成熟儿 OR SU=婴儿 OR SU=低出生体重儿 OR SU=低体重儿 OR SU=极低出生体重儿 OR SU=极低体重儿 OR SU=超低出生体重儿 OR SU=超低体重儿 OR SU=低出生体质量儿 OR SU=低体质量儿 OR SU=极低出生体质量儿 OR SU=极低体质量儿 OR SU=超低出生体质量儿 OR SU=超低体质量儿

#2 SU=乳铁蛋白 OR SU=乳铁传递蛋白 OR SU=乳铁转铁蛋白

#3 #1 AND #2

**Wanfang search strategy:**

#1 主题:(出生时低体重) + 主题:(早产儿) + 主题:(新生儿) + 主题:(未成熟儿) + 主题:(婴儿) + 主题:(低出生体重儿) + 主题:(低体重儿) + 主题:(极低出生体重儿) + 主题:(极低体重儿) + 主题:(超低出生体重儿) + 主题:(超低体重儿) + 主题:(低出生体质量儿) + 主题:(低体质量儿) + 主题:(极低出生体质量儿) + 主题:(极低体质量儿) + 主题:(超低出生体质量儿) + 主题:(超低体质量儿)

#2 主题:(乳铁蛋白) + 主题:(乳铁传递蛋白) + 主题:(乳铁转铁蛋白)

#3 #1 AND #2
