# Supplementary figures and images for: Enteral Lactoferrin Supplementation for Preventing Sepsis and Necrotizing Enterocolitis in Preterm Infants: A Meta‑Analysis With Trial Sequential Analysis of Randomized Controlled Trials
Source: Front Pharmacol. 2020 Aug 7;11:1186. doi: 10.3389/fphar.2020.01186 (PMC7426497; doi:10.3389/fphar.2020.01186)

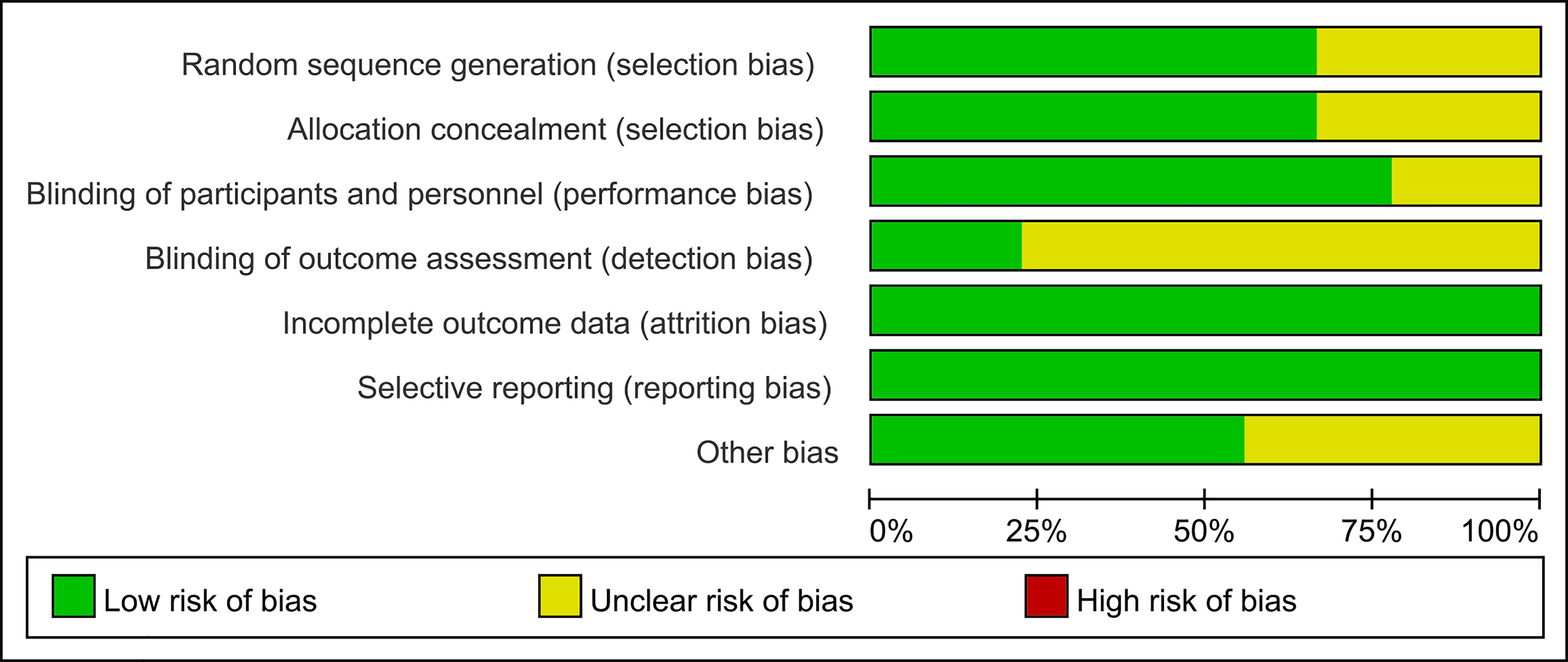

Supplement: Figure S1 — Risk of bias about each risk of bias item resented as percentages across all included studies. (TIF 2356 kb). [file Image_1.tif]

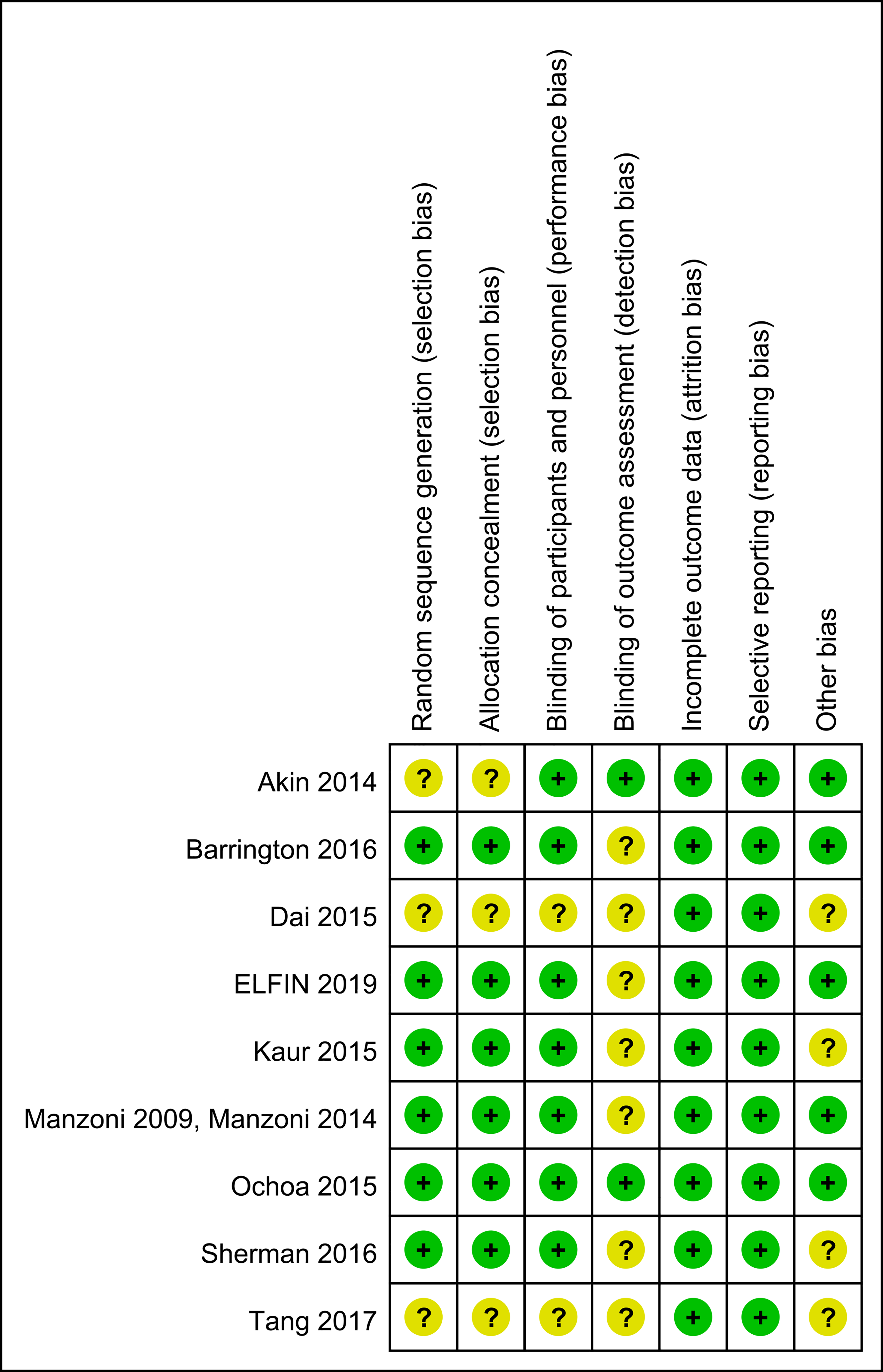

Supplement: Figure S2 — Risk of bias summary for each included study. Yellow ()? indicates unclear risk; and green (+) indicates low risk of bias. (TIF 5643 kb). [file Image_2.tif]

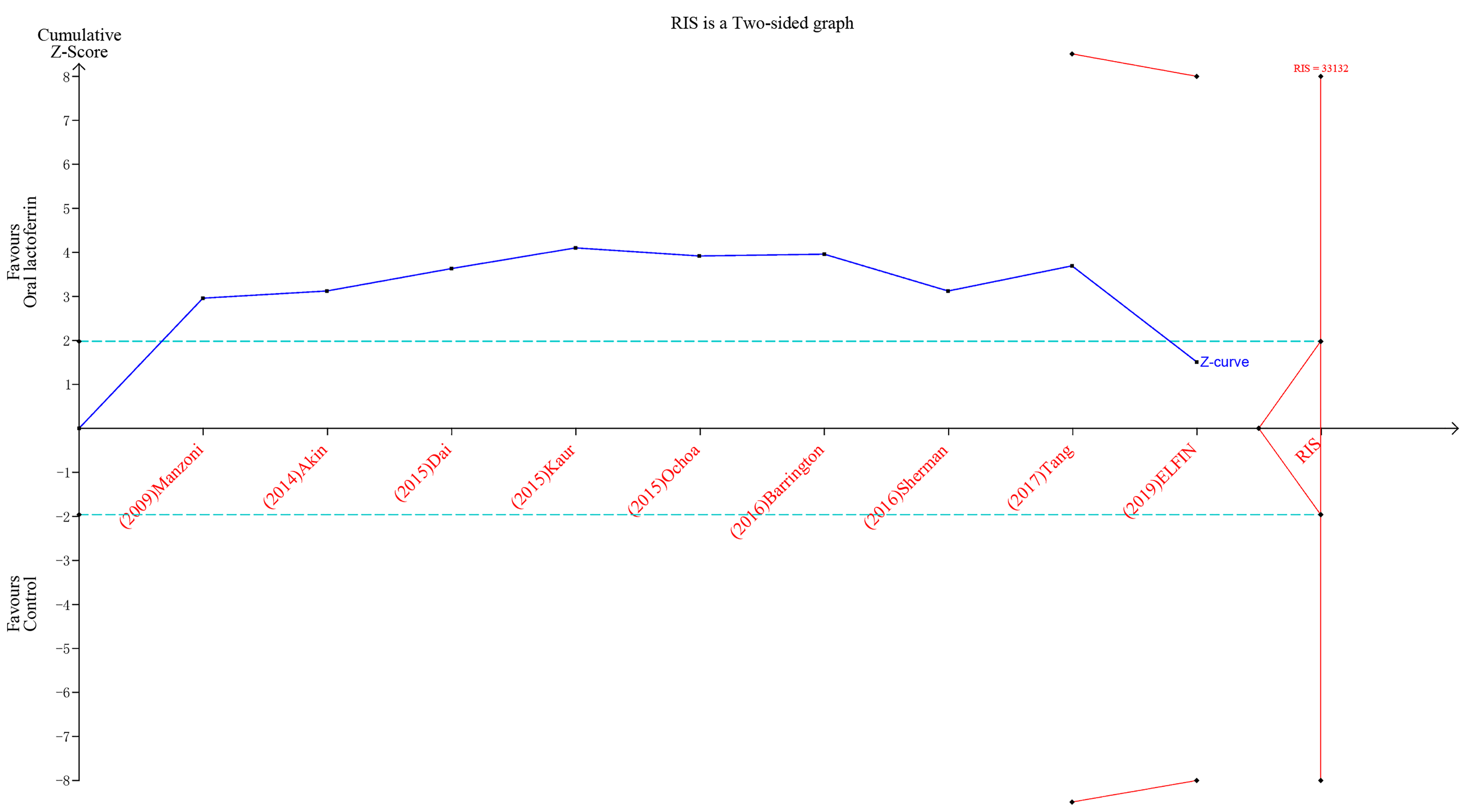

Supplement: Figure S3 — Trial sequential analysis on pooled results of nine trials comparing enteral lactoferrin with placebo for the prevention of sepsis. Trial sequential analysis indicating that the cumulative z curve (blue) crossed the conventional line, but reached neither the trial sequential monitoring boundary for benefit nor the estimated information size boundary. A diversity-adjusted required information size of 33132 patients was calculated using α=0.05 (two-sided) and β=20 (power 80%), an anticipated relative risk reduction of 20%, and an event proportion of 16.01% in the control arm. (TIF 1444 kb). [file Image_3.tif]

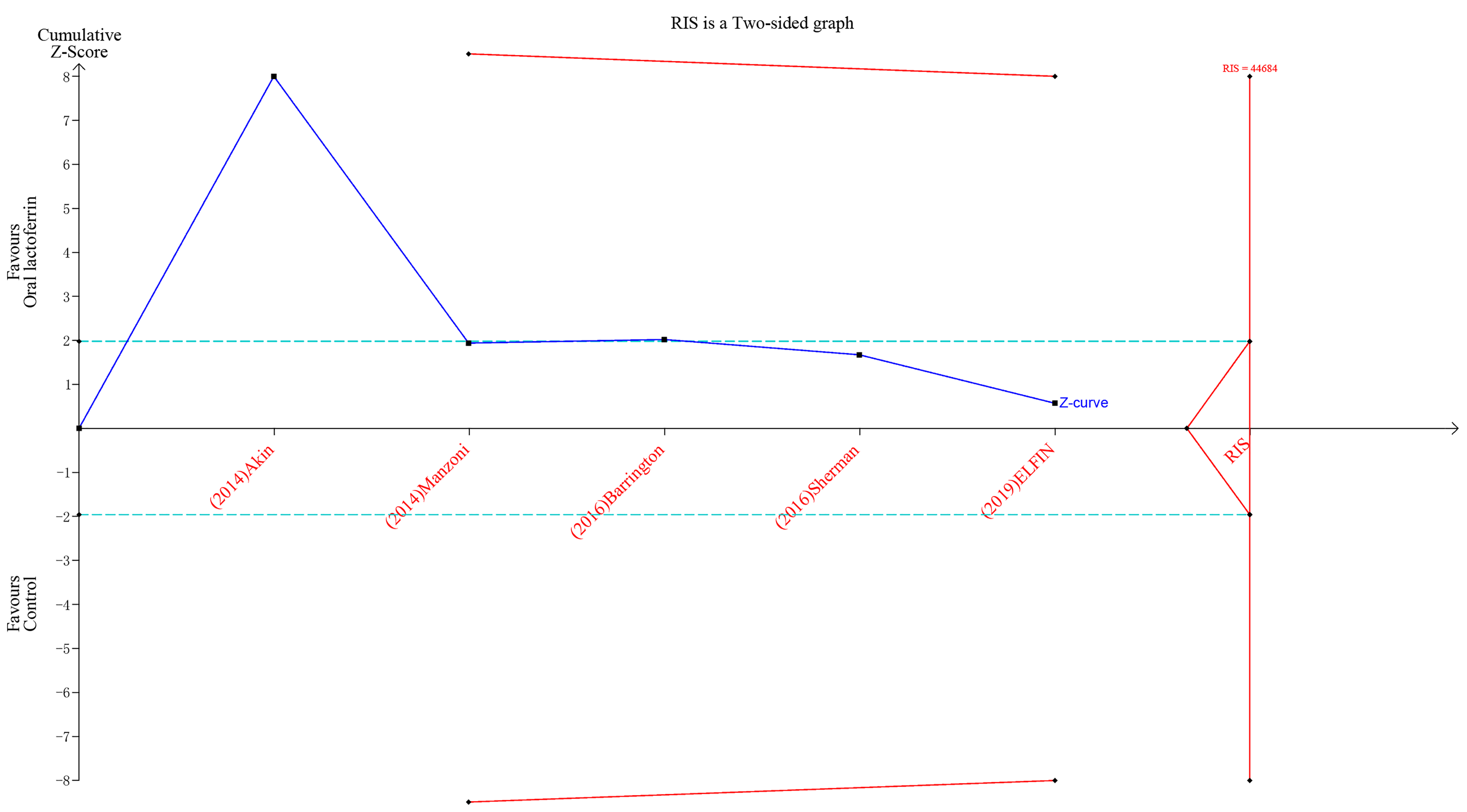

Supplement: Figure S4 — Trial sequential analysis on pooled results of five trials comparing enteral lactoferrin with placebo for prevention of Necrotizing Enterocolitis (NEC) Bell’s stage II or III. Trial sequential analysis indicating that the cumulative z curve (blue) crossed the conventional line, but reached neither the trial sequential monitoring boundary for benefit nor the estimated information size boundary. A diversity-adjusted required information size of 44684 patients was calculated using α=0.05 (two-sided) and β=20 (power 80%), an anticipated relative risk reduction of 20%, and an event proportion of 5.32% in the control arm. (TIF 1414 kb). [file Image_4.tif]

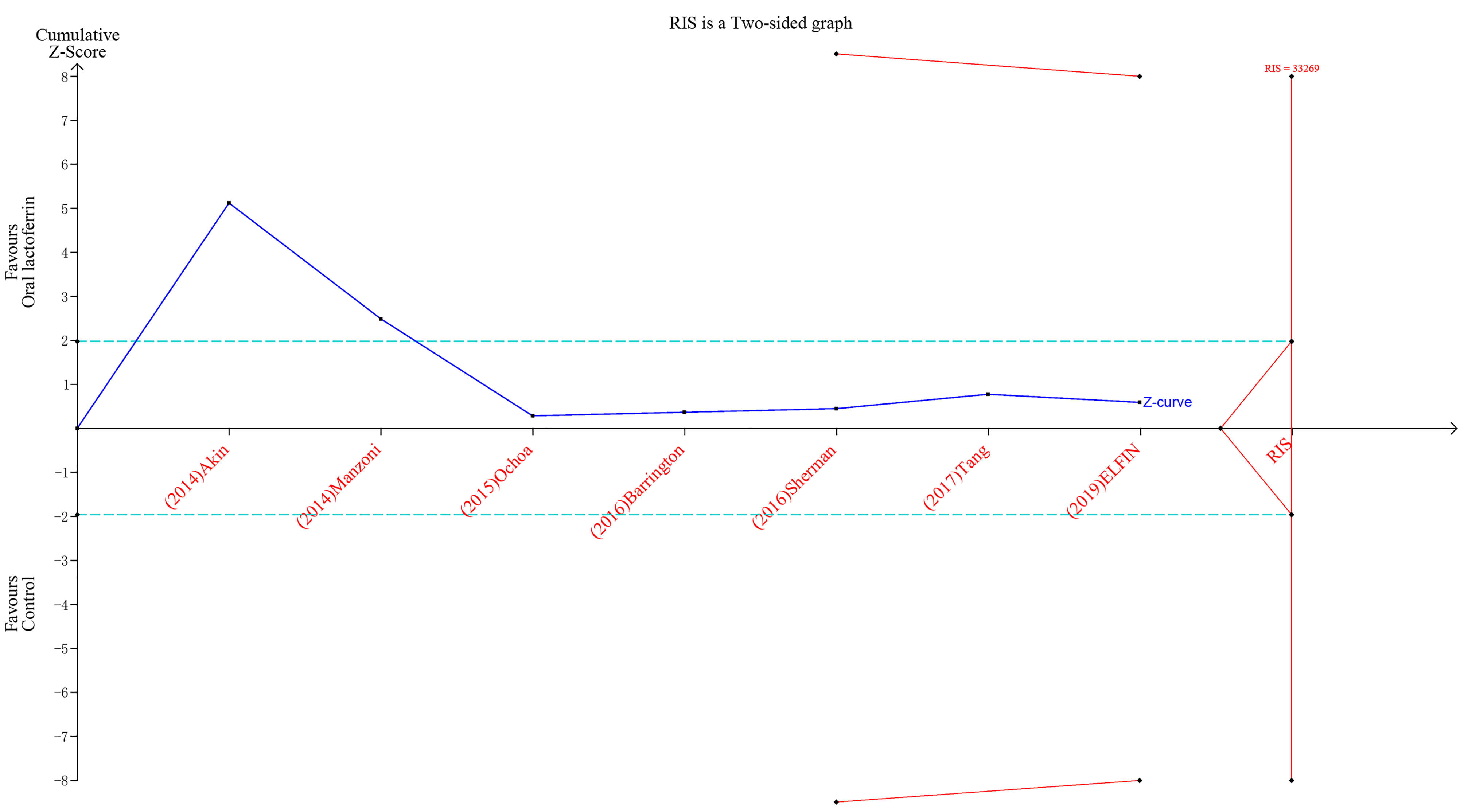

Supplement: Figure S5 — Trial sequential analysis on pooled results of seven trials comparing enteral lactoferrin with placebo for reducing all-cause mortality. Trial sequential analysis indicating that the cumulative z curve (blue) crossed the conventional line, but reached neither the trial sequential monitoring boundary for benefit nor the estimated information size boundary. A diversity-adjusted required information size of 33269 patients was calculated using α=0.05 (two-sided) and β=20 (power 80%), an anticipated relative risk reduction of 20%, and an event proportion of 6.09% in the control arm. (TIF 1424 kb). [file Image_5.tif]

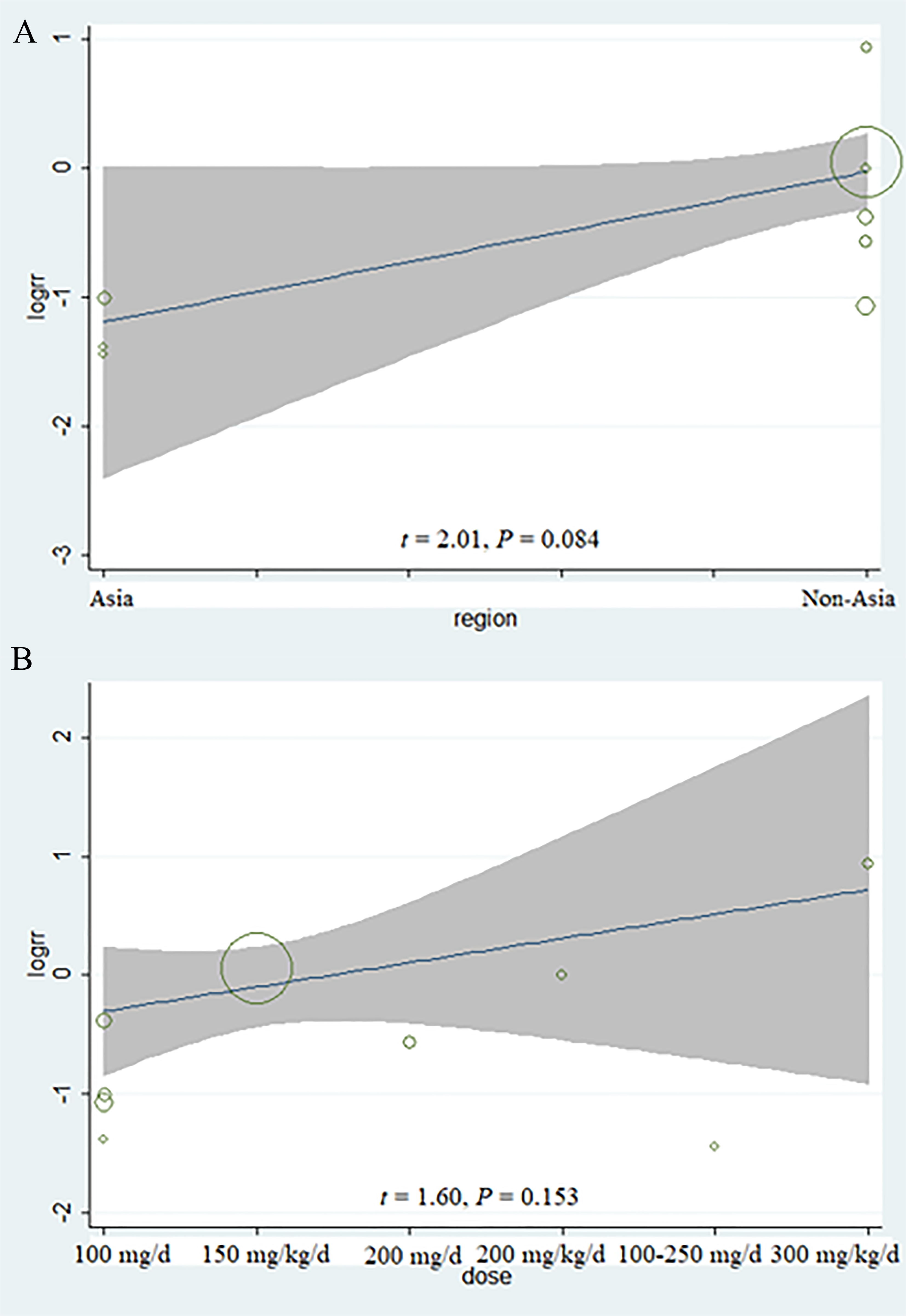

Supplement: Figure S6 — Univariate meta-regression analysis on the (A) region of patients, (B) dose of lactoferrin for the outcome of late-onset sepsis. (TIF 7069 kb). [file Image_6.tif]

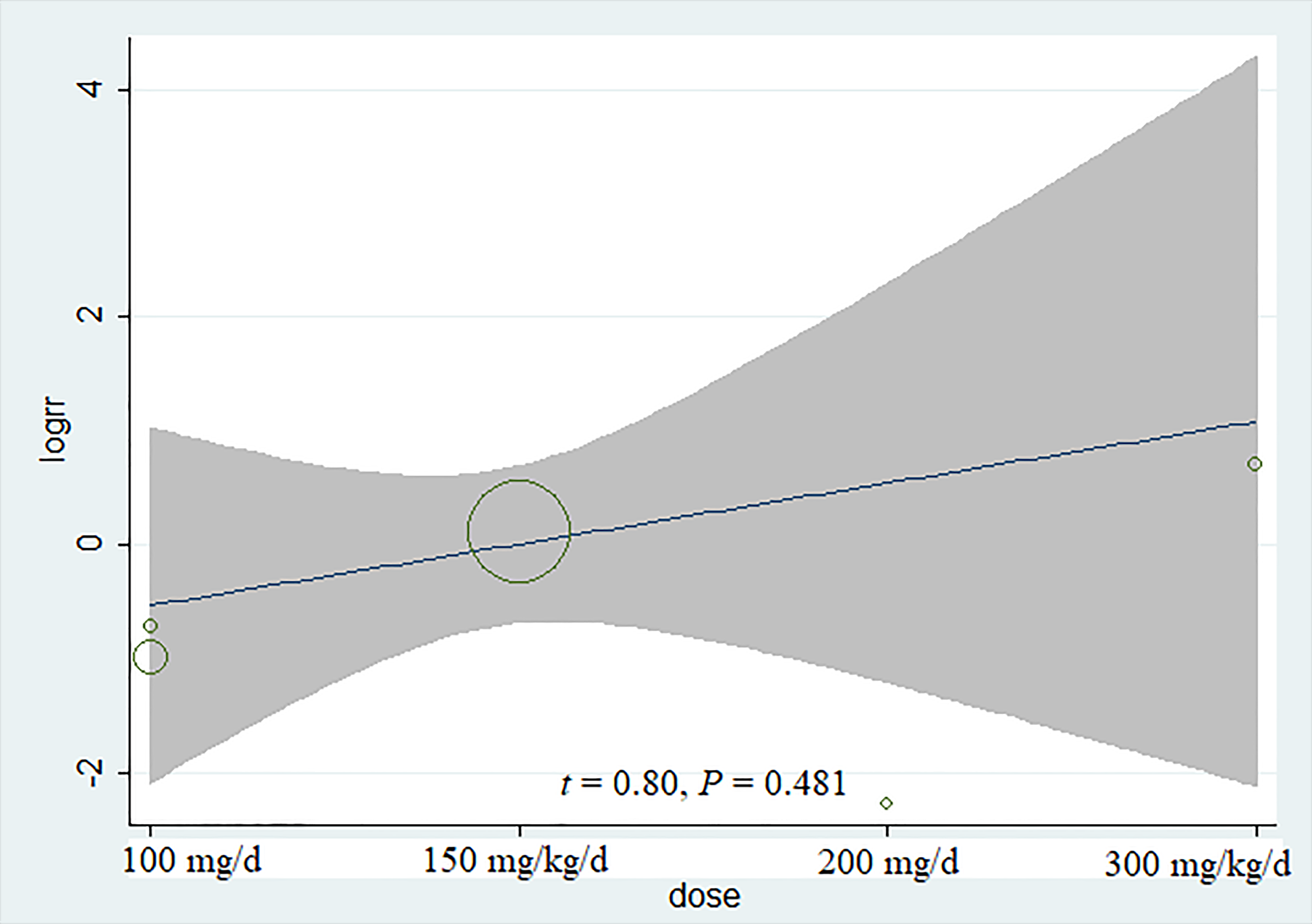

Supplement: Figure S7 — Univariate meta-regression analysis on the dose of lactoferrin for the outcome of NEC stage II or III. (TIF 2950 kb). [file Image_7.tif]

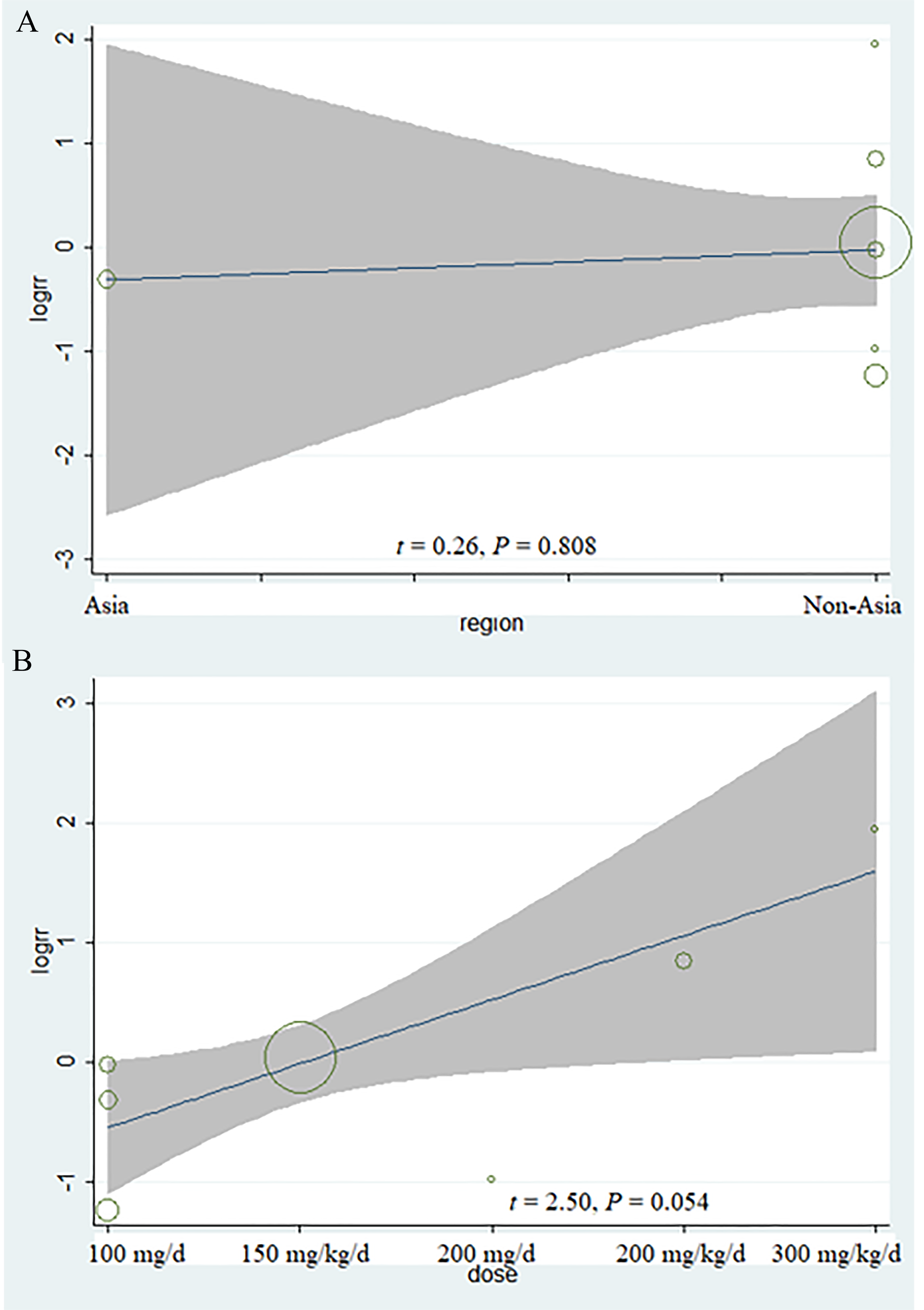

Supplement: Figure S8 — Univariate meta-regression analysis on the (A) region of patients, (B) dose of lactoferrin for the outcome of all-cause mortality. (TIF 7005 kb). [file Image_8.tif]
